# Risk Factors for Glenohumeral Internal Rotation Deficit in Adolescent Athletes: A Comparison of Overhead Sports and Non-overhead Sports

**DOI:** 10.7759/cureus.34045

**Published:** 2023-01-21

**Authors:** Kentaro Ohuchi, Hiroaki Kijima, Hidetomo Saito, Yusuke Sugimura, Takayuki Yoshikawa, Naohisa Miyakoshi

**Affiliations:** 1 Orthopaedic Surgery, Yokote Municipal Hospital, Yokote, JPN; 2 Orthopaedic Surgery, Akita University Graduate School of Medicine, Akita, JPN; 3 Orthopaedic Surgery, Akita Rosai Hospital, Oodate, JPN; 4 Orthopaedic Surgery, Oomagari Welfare Medical Center, Daisen, JPN

**Keywords:** overhead motions, medical checks, overhead sports, adolescent athletes, glenohumeral internal rotation deficit

## Abstract

Background

A glenohumeral internal rotation deficit (GIRD) occurs in baseball players due to the repetitive pitching motion. However, few reports have addressed associations between GIRD and sports other than baseball. In this study, we investigated whether GIRD occurs in adolescent athletes playing overhead sports other than baseball, and also, the risk factors that cause GIRD in these sports were examined.

Methods

A total of 214 junior high school athletes who had undergone medical checks were evaluated. Seventy-five athletes playing sports requiring overhead motions were classified into the overhead sports group (39 tennis, 18 handball, 12 badminton, and 6 softball players). Eighty athletes participating in sports requiring the use of the upper limbs but not requiring frequent overhead motions were classified into the non-overhead sports group (31 kendo, 20 fencing, 19 basketball, and 10 table tennis players); 59 athletes who mainly did not use an upper limb were classified into the contact sports group (22 judo, 15 wrestling, 13 soccer, and 9 rugby football players). The range of shoulder motion (internal rotation, external rotation, and total arc), background factors, general laxity, and flexibility of the lower body were compared among the three groups.

Results

Thirty-four (16%) of 214 players were classified as having GIRD (internal rotation deficit >15°). Significantly more athletes had GIRD in the overhead sports group than in the other groups (p=0.007). The internal rotation deficit was significantly worse in the overhead sports group than in the other groups (p=0.006, p=0.02, respectively). Background factors, general laxity, and lower body flexibility did not differ significantly among the groups.

Conclusion

The sole risk factor for GIRD was participating in any sport that required overhead movements. Thus, not only baseball players, but also other athletes who participate in sports requiring overhead movements should receive correct information to prevent GIRD.

## Introduction

A glenohumeral internal rotation deficit (GIRD) is defined as a decrease in the internal rotation range of the dominant shoulder compared with the non-dominant shoulder [1,2]. GIRD is triggered by repetitive overhead throwing motion, and the reduction in the internal rotation range is a risk factor for shoulder injuries in baseball players [3,4]. Furthermore, in sports that require raising the hand up over the head, overhead sports such as tennis or handball, there have been many reports of GIRD [5-7]. However, the subjects of these reports were adult athletes, and there have been a few reports of GIRD in adolescent athletes playing overhead sports.

If GIRD were found in adolescent athletes other than baseball players, it might be considered a risk factor for shoulder injuries in overhead sports players. Thus, the purpose of this study was to investigate whether GIRD occurs in adolescent athletes playing overhead sports other than baseball and to clarify the risk factors for GIRD in these sports.

## Materials and methods

A total of 214 junior high school students designated by the prefectural physical education association who received comprehensive examinations were considered for the study. Approval for this study was granted by the institutional review board of our university (Certified Clinical Research Review Board, Akita University, approval number 1704), and all subjects gave their informed consent to participate.

The subjects were divided into three categories: overhead sports group (total 75 players: 39 tennis players, 18 handball players, 12 badminton players, and 6 softball players), who raise the hand up over the head; the non-overhead sports group (total 80 players: 31 kendo players, 20 fencers, 19 basketball players, and 10 table tennis players), who mainly use one upper limb, but do not need to raise the hand up over the head; and the contact sports group (total 59 players: 22 judo players, 15 wrestlers, 13 soccer players, and 9 rugby football players), who do not primarily use the upper limb.

During the examinations, the range of motion of the shoulder was assessed in the supine position with a goniometer. In particular, internal rotation and external rotation range at 90° of shoulder abduction were measured, and their sum was defined as the total arc. Then, GIRD was defined as a dominant-nondominant side difference greater than 15° in the range of internal rotation of the shoulder, and the incidence of GIRD and the range of motion of the shoulder were compared among the three groups [8,9]. In addition, the heel-buttock distance, straight leg raising angle, range of motion of the hip joint, and the presence of joint laxity were compared between the players with GIRD and those without. The subjects were also asked how many years they had been playing the sports, and how much their height had increased over the past year.

For statistical analysis, the chi-squared test was used to compare the incidence of GIRD, and the unpaired t-test was used to compare other factors between the players with and without GIRD. Significance was set at p<0.05.

## Results

Thirty-four (16%) of 214 players were estimated to have GIRD. The rate of GIRD was 27% (20 of 75) in the overhead sports group, 10% (8 of 80) in the non-overhead sports group, and 10% (6 of 59) in the contact sports group; the rate of GIRD was significantly higher in the overhead sports group (p=0.007) (Table 1). The dominant-nondominant side differences in the internal rotation range and of the total arc of the shoulder in the overhead sports group were 9.1° and 8.7°, respectively, which were significantly higher than those in the other groups (Table 2). Average age, average years of competition, and height growth in the past year were not significantly different between the GIRD-positive and GIRD-negative groups (Table 3). There were no significant differences between the GIRD-positive and GIRD-negative group in items related to lower body flexibility (Table 4).

**Table 1:** Rate of GIRD in study groups GIRD, glenohumeral internal rotation deficit The rate of GIRD was significantly higher in the overhead sports group (p=0.007).

	Positive GIRD	Negative GIRD	Rate of GIRD
Overhead sports group (n = 75)	20	55	27%
Non-overhead sports group (n = 80)	8	72	10%
Contact sports group (n = 59)	6	53	10%
Total (n = 214)	34	180	16%

**Table 2:** Range of motion of the shoulder joint ^a^p = 0.006 versus non-overhead sports group ^b^p = 0.02 versus collision sports group ^c^p = 0.001 versus non-overhead sports group ^d^p = 0.006 versus collision sports group

	Overhead sports group (n = 75)	Non-overhead sports group (n = 80)	Contact sports group (n = 59)
Internal rotation of the dominant arm (°)	59.3 ± 15.1	63.9 ± 13.4	64.7 ± 11.9
Internal rotation of the non-dominant arm (°)	66.8 ± 14.0	66.5 ± 14.0	67.4 ± 11.9
Difference in internal rotation (°)	9.1 ± 9.6^a,b^	3.4 ± 10.0	3.7 ± 9.4
External rotation of the dominant arm (°)	97.1 ± 8.6	98.3 ± 9.2	94.2 ± 7.9
External rotation of the non-dominant arm (°)	96.9 ± 10.4	96.4 ± 9.2	93.0 ± 5.8
Difference in external rotation (°)	-0.3 ± 8.5	-3.0 ± 6.7	-2.5 ± 6.9
Total arc of the dominant arm (°)	156.3 ± 19.1	162.3 ± 17.0	158.9 ± 14.4
Total arc of the non-dominant arm (°)	163.7 ± 20.3	162.9 ± 16.5	160.4 ± 13.1
Difference in the total arc (°)	8.7 ± 11.4^c,d^	0.7 ± 10.8	1.8 ± 10.1

**Table 3:** Background factors GIRD, glenohumeral internal rotation deficit Average age, average years of competition, height growth in the past year did not show any significant difference between the GIRD-positive and the GIRD-negative groups.

	Positive GIRD (n = 34)	Negative GIRD (n = 180)	p value
Age (years)	14.0 ± 0.8	13.7 ± 1.3	0.104
Competition years of sports (years)	6.1 ± 2.0	6.3 ± 2.0	0.629
Height growth in the past year (cm)	3.9 ± 2.6	4.8 ± 2.9	0.077

**Table 4:** Lower body flexibility estimates GIRD, glenohumeral internal rotation deficit There were no significant differences between the GIRD-positive and GIRD-negative groups for items relating to lower body flexibility.

	Positive GIRD (n = 34)	Negative GIRD (n = 180)	p value
Heel-buttock distance (cm)	3.5 ± 5.0	4.0 ± 5.3	0.608
Right straight leg raising angle (°)	74.6 ± 13.4	74.1 ± 14.0	0.855
Left straight leg raising angle (°)	75.6 ± 13.4	73.9 ± 14.3	0.529
Internal rotation of the right hip (°)	44.3 ± 21.6	40.6 ± 18.2	0.301
Internal rotation of the left hip (°)	46.5 ± 16.5	40.5 ± 16.8	0.068
External rotation of the right hip (°)	60.9 ± 14.0	56.9 ± 13.8	0.124
External rotation of the left hip (°)	59.9 ± 15.0	56.1 ± 13.0	0.133

## Discussion

There are mainly two factors that affect the change in the range of motion of the pitching side shoulder: factors related to soft tissue, which include decreased flexibility of the posterior joint capsule or muscles, laxity of the anterior soft tissue, and tightness of the muscles around the shoulder joint; another factor is bone derived that is an increase in the posterior torsion of the humeral head. These factors affect the changes in the range of motion of the pitching side shoulder of baseball players [10,11].

There are some reports about changes in the range of motion of the shoulder in sports other than baseball [12,13]. However, these reports include adults, and there are only a few reports of adolescent athletes involved in overhead sports other than baseball. To our knowledge, this study is the first to compare overhead sports other than baseball and non-overhead sports in adolescent athletes. The results of the present study clarified that GIRD does not occur only by repeated use of the upper limbs, and that repetition of overhead throw motion similar to pitching motion causes GIRD. In addition, it was suggested that GIRD may occur even in adolescent athletes involved in overhead sports other than baseball.

In the present study, there was no significant difference in the external rotation range among the three groups. In the previous studies on baseball, the external rotation range of the shoulder of the pitching side was reported to increase since the early phase [14,15]. As one of the reasons why the external rotation range was not increased in the overhead sports group in this study, it can be considered that the posterior torsion angle of the humeral head was small because the athletes were relatively young. It was also possible that the overhead throw motion in the overhead sports group in the present study did not cause excessive external rotation of the shoulder during the acceleration phase as much as the pitching motion in baseball.

In the present study, no significant differences were found in background factors between the GIRD-positive and GIRD-negative groups. From these results, it can be deduced that the change in the range of motion of the shoulder may occur regardless of the age when the overhead sports were started or the duration of time since the players started overhead sports. Levine et al. in a study divided 298 baseball players into three groups according to age and investigated changes in the range of motion of the shoulder. They found that the range of internal rotation of the dominant shoulder already decreased in the young group of 8- to 12-year-olds [15]. Thus, the athletes who perform overhead sports may develop GIRD from the early stage in any sport.

There was also no significant difference in lower body flexibility between the GIRD-positive and GIRD-negative groups. There are some reports about the relationship between the range of motion of the shoulder and lower body tightness in baseball players [16-18]. However, pitching-like motions in the overhead sports group in the present study (for example, serve or smash) are often performed without a kinetic chain mechanism that starts from the lower body and transfers kinetic energy to the upper limbs, such as the pitching motion in baseball; thus, there was no clear difference in lower body flexibility in the current study. In other words, the pitching-like motion in the overhead sports group may not be affected by lower body flexibility as much as the pitching motion in baseball. This is also one of the new findings of this research. In addition, a decrease in the range of internal rotation of the shoulder causes not only shoulder pain, but also a decrease in competitive performance [19,20]. From the perspective of prevention of sports injuries, it is necessary to improve shoulder range of motion in adolescent athletes.

The limitation of this study is that the relationship between the change in the range of motion and shoulder pain or performance in competition was not examined. In the future, the effect on competitive performance should be evaluated by a survey of the relationship between changes in the range of motion and pain in the shoulder through a prospective, interventional study. To our knowledge, this study provided, for the first time, the important evidence leading to such future research about the prevention of sports injuries.

## Conclusions

The rate of GIRD was significantly higher in overhead sports players than in other players. There were no significant differences in physical characteristics and background factors between the GIRD-positive group and the GIRD-negative group. Therefore, playing overhead sports, in which an action similar to the overhead pitching motion is repeated, was the risk factor for GIRD. In other words, any athlete who plays overhead sports may develop GIRD. Thus, appropriate prevention or early intervention may be necessary not only for baseball players, but also for all overhead sports players.
